# A102 NOVICE ENDOSCOPISTS EXPERIENCE AND PERFORMANCE ON A NOVEL PHYSICAL-COMPUTER COLONOSCOPY SIMULATOR

**DOI:** 10.1093/jcag/gwae059.102

**Published:** 2025-02-10

**Authors:** A Almneni, N Gimpaya, W Tran, D Tham, A Wiggins, A Ibrahim, R Sondawle, G Sinanian, J Lisondra, F Dang, J Samarasena, S Grover

**Affiliations:** Royal College of Surgeons in Ireland, Dublin, Ireland; Scarborough Health Network, Scarborough, ON, Canada; Education Research, Scarborough Health Network, Scarborough, ON, Canada; Scarborough Health Network, Scarborough, ON, Canada; Scarborough Health Network, Scarborough, ON, Canada; Scarborough Health Network, Scarborough, ON, Canada; Scarborough Health Network, Scarborough, ON, Canada; Scarborough Health Network, Scarborough, ON, Canada; Scarborough Health Network, Scarborough, ON, Canada; University of California Irvine, Irvine, CA; University of California Irvine, Irvine, CA; Scarborough Health Network, Scarborough, ON, Canada

## Abstract

**Background:**

Simulation-based training mitigates safety risks for patients in the early stages of endoscopic training. The MIKOTO colonoscopy simulator is a hybrid physical-computer model that assesses forces applied during simulated procedures via sensor-generated feedback to the user.

**Aims:**

To describe the technical performance and self-reported experience of novice endoscopists when using the MIKOTO.

**Methods:**

24 novices attended a simulation course in Toronto which included activities on benchtop, colon phantoms, and the MIKOTO. Trainees completed MIKOTO’s easiest case in 10 minutes. The primary outcome measure was the self-reported user experience using a 5-point Likert scale. Other outcomes included relevant DOPS items rated by an expert, and a MIKOTO-generated score integrating colon elongation and completion time.

**Results:**

11 Gastroenterology and 13 General Surgery novices (<500 procedures) attended. The median rating for “feels like the low-fidelity (wooden simulator)” was 2 with an IQR of 2 (Table 1). The median rating for “looks like performing colonoscopy on a live patient” was 4 with an IQR of 2. The median score for “Further use of the Mikoto will help improve my overall colonoscopy performance” was 5. The median ratings on relevant DOPS items ranged from 1 to 2. The median MIKOTO score was 27.5 with an interquartile range (IQR) of 5.

**Conclusions:**

The expert-assessed DOPS rating and Mikoto-generated scores indicate the ability of the Mikoto to show the skill level of the novice trainees. Self-reported experiences indicate similarity of visual and haptic realism compared to live procedures. The MIKOTO, however, was distinct from the low-fidelity benchtop simulator. A limitation of this study is the brief time spent on the simulator and the lack of orientation to the MIKOTO. Further studies are needed to compare technical performance and subjective experience between novices, intermediate and expert endoscopists. Different forms of validity must be investigated. The experience of the novice endoscopists provide insight on future integration of the MIKOTO into training programs.

Table 1. Mikoto Experience Form

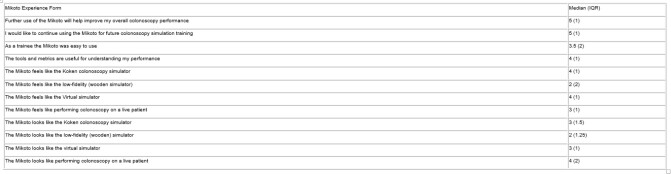

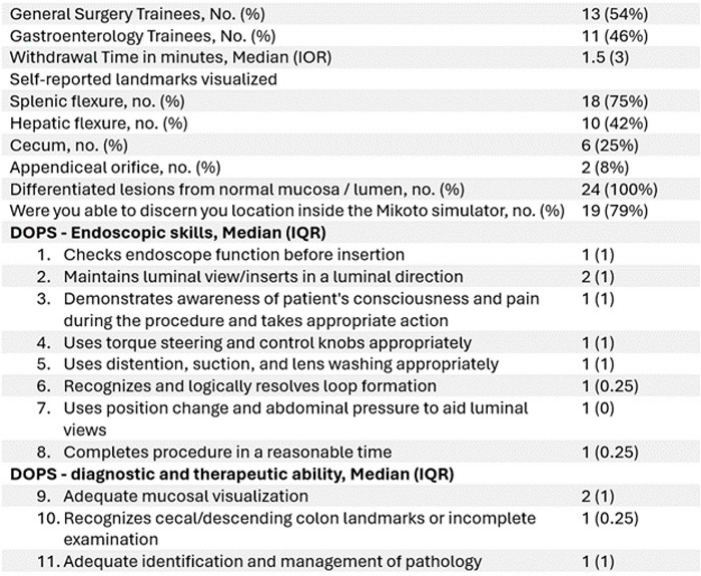

Demographics and DOPS Ratings

**Funding Agencies:**

None

